# Simulation of blast lung injury induced by shock waves of five distances based on finite element modeling of a three-dimensional rat

**DOI:** 10.1038/s41598-019-40176-7

**Published:** 2019-03-05

**Authors:** Chang Yang, Zhang Dong-hai, Liu Ling-ying, Yu Yong-hui, Wu Yang, Zang Li-wei, Han Rui-guo, Chai Jia-ke

**Affiliations:** 1 Department of Burn and Plastic Surgery, Burns Institute, Burn & Plastic Hospital of PLA General Hospital, Fourth Medical Center of PLA General Hospital, Beijing, 100048 PR China; 2 Science and Technology on Transient Impact Laboratory, Beijing, 102202 PR China

## Abstract

Blast lung injury (BLI) caused by both military and civilian explosions has become the main cause of death for blast injury patients. By building three-dimensional (3D) models of rat explosion regions, we simulated the surface pressure of the skin and lung. The pressure distributions were performed at 5 distances from the detonation center to the center of the rat. When the distances were 40 cm, 50 cm, 60 cm, 70 cm and 80 cm, the maximum pressure of the body surface were 634.77kPa, 362.46kPa, 248.11kPa, 182.13kPa and 109.29kPa and the surfaces lung pressure ranges were 928–2916 Pa, 733–2254 Pa, 488–1236 Pa, 357–1189 Pa and 314–992 Pa. After setting 6 virtual points placed on the surface of each lung lobe model, simulated pressure measurement and corresponding pathological autopsies were then conducted to validate the accuracy of the modeling. For the both sides of the lung, when the distance were 40 cm, 50 cm and 60 cm, the Pearson’s values showed strong correlations. When the distances were 70 cm and 80 cm, the Pearson’s values showed weak linear correlations. This computational simulation provided dynamic anatomy as well as functional and biomechanical information.

## Introduction

As a result of the continuous growth of conflicts and urban terrorism, deaths caused by explosions still occur in some areas^1^. Terrorist explosive worldwide events have risen since 1970 and increased rapidly after 2000^2^. An explosion can produce a shock wave - the pressure of the shock wave rises rapidly to form overpressure or peak pressure and then rapidly decline to form negative pressure^1^. Overpressure and negative pressure are the primary causes of primary blast injuries, mainly inducing damage in gas-containing organs such as lungs, the gastrointestinal tract and auditory organs^3^. Blast lung injury (BLI) caused by both military and civilian explosions has become the main cause of death for blast injury patients^4^.

The lungs are easily affected by shock waves because they are air-containing^5^. Due to overpressure, the pulmonary capillaries are expanded and moved instantaneously. The overpressure also causes alveolar wall and capillary rupture^6^. Alveolar wall rupture directly affects lung ventilation, resulting in insufficient ventilation, which then causes systemic hypoxia^7^. After the rupture of the capillaries, the blood leaks and the blood flow in the lungs decreases, leading to pulmonary ischemia and hypoxia^8^. Tsokos *et al*. found in 8 autopsy cases with blast lung injuries that 8 patients suffered lung collapse, ruptured lung tissues, bullae and pulmonary hemorrhage. 4 cases had gas embolism and 4 had fat embolism, while 3 patients had bone marrow embolism^9^. Chang *et al*. used density analysis based on micro-CT to investigate lung injuries in terms of blast injuries combined with burn injuries and found that lung tissue density was much higher in blast injuries than in cases with no injury^10^.

In order to investigate the characteristics of a blast injury, shock tubes were invented in the 1950s^11^. Jaffin *et al*. then developed a smaller shock tube in order to perform animal experiments in 1987. In 1988, a biological shock tube was developed through research in China to generate blast injury^12^. This instrument could release compressed air to form a high-speed pressure wave including both overpressure and underpressure^11^. However, the explosion characteristics including pressure and the elapsed time of the tube were still different from traditional explosion devices. A moderate blast injury on rats 50 cm away from the source of the explosion and a full-thickness burn injury of 30% total body surface area were both conducted to establish a burn-blast combined injury model^10^. The rats with a combined injury had severe lung injuries.

Neither clinical studies nor animal research studies have fully explained lung injuries from blast shock waves. Traditional explosive substances are susceptible to wind direction, environment, temperature and the location of the body. In animal experiments, sensors can only be placed in a few observation points and whole pressure distribution cannot be observed completely. In comparison to computational simulations, explosion devices need skilled operators, explosion substances and an specialized experiment place.

Finite element (FE) modeling and simulations have been used to predict the responses of biological structures under various mechanical conditions, including air and liquid flow conditions, transmission and pressure distribution of blast wave. This approach could provide us an increasing reliability and more detailed biomechanical information based on distribution diagram and statistics, which might be helpful in further predictive analysis of injuries^13,14^. Researchers have used this computational modeling to investigate the pressure distribution of blast injuries^13,15^. Wang *et al*. simulated blast waves at five blast intensities from the anterior, right lateral and posterior directions one meter from the detonation center and found that the intracranial pressure wave went through the posterior fossa and vertebral column, causing high pressure levels^15^. Zhang *et al*. evaluated the response of the head to blast loadings with and without a helmet with FE modeling^16^. Some researchers have used finite element research for thorax deformation to investigate breathing movement^17^.

However, few researchers have used this technique to investigate blast lung injury. In order to observe blast lung injury more accurately and reliably, we implemented finite element modeling to mimic blast lung injury. By building three-dimensional (3D) models of rat explosion regions, we simulated the surface pressure of the skin and lung. The pressure distributions were performed at 5 distances from the detonation center to the center of the rat. Pressure measurement and pathological autopsies were then conducted to validate the accuracy of the modeling.

## Methods

### Finite element 3D rat modeling

The 3D rat model was developed from a series of CT images of a small animal micro-CT (Siemens Medical Solutions, Knoxville, TN). The size of the rat (excluding the limbs) was 20.11 cm long, 10.32 cm wide and 10.04 cm tall. By using MIMICS software (Mimics 17.0, Materialise, Leuven, Belgium), CT images from the rat were all combined as one 3D structure. After setting the CT threshold range and reconstruction area, all structural data was defined to approach the actual components. The geometric model included linear tetrahedral elements, hexahedral elements and pyramid elements for 3D construction. The 3D model was then segmented into the lung, heart, rib cage, skin and muscle components. ANSYS ICEM CFD 14.5 (ANSYS, Inc., Canonsburg, PA) was used for meshing highly complex geometry into a high-quality grid in order to calculate the pressure levels. The fully developed finite element rat model grids included the 3D finite element model grids of the lung, heart, rib cage, skin and muscle layers.

**Figure 1:**
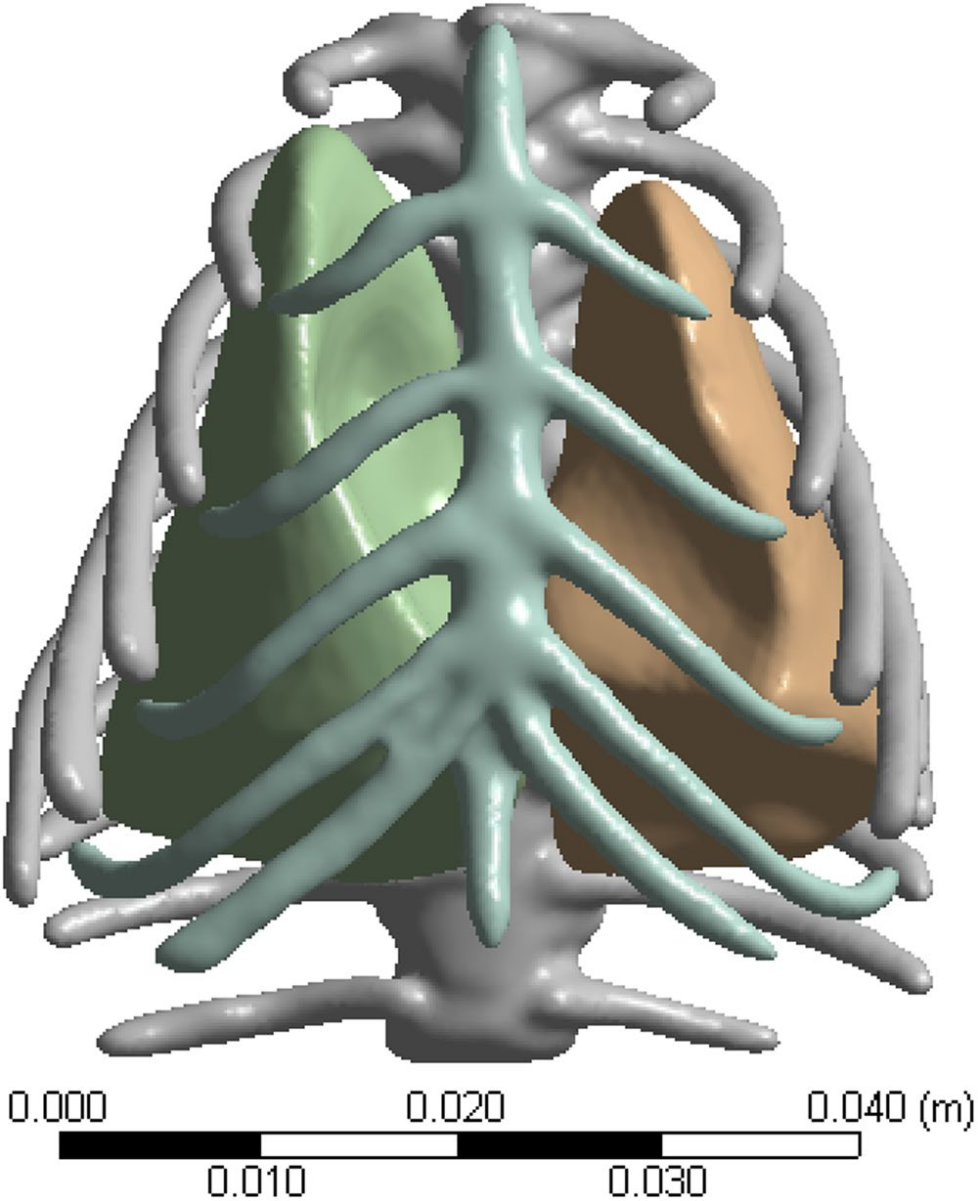
3D finite element models of the lung and rib cage.

The whole model consists of 3,960,894 elements including the whole lung (780,382 elements), the whole heart (338,281 elements), the whole rib cage (641,293 elements), and the whole skin and muscle (2,200,938 elements) (Fig. 1).

### Material modeling

The material constants of the components in the body were chosen from literature. The lungs are pyramid-shaped, paired organs attached to the trachea by the right and bronchi that are left on the inferior surface. Each lung has smaller units called lobes. Each lobe is composed of multiple bronchopulmonary segments. The bronchioles eventually end in clusters of microscopic air sacs called alveoli. Between the alveoli is a thin layer of cells called the interstitium, which contains blood vessels and cells that help support the alveoli^18^. In this simulation, since the lung tissue was mostly composed of gas-filled alveoli, the lung tissue could be approximated as an elastic structure. According to the measurement of the lung’s elastic modulus, this constant was set at 1.2 × 10^4^ Pa and the Poisson’s ratio was 0.32^19^. An elastic modulus is a quantity that measures an object or substance’s resistance to being deformed elastically. Poisson’s ratio is the ratio of relative contraction to relative expansion, also a measure of the Poisson effect, which means a material tends to expand in directions perpendicular to the direction of compression^19–21^.

The rib cage is composed of bone material. According to the research by Ladd AJ, after a combination of mechanical testing, three-dimensional imaging and finite-element modeling, 6.6 GPa was selected as the elastic modulus of the rib material in this simulation. Poisson’s ratio was set at 0.3^21^.

In the 3D construction process, the skin layer was too thin to be distinguished from the muscles and soft tissue. The skin and muscle layers were combined as one layer in this simulation. The heart is composed of myocardial tissue and valve structures. In CT scanning, the heart tissue density was almost the same as the skeletal muscle. Therefore, we combined the skin layer, structure of heart and muscle as the same material in this simulation with an elastic modulus of 480 MPa and Poisson’s ratio 0.45^22^.

### Establishing the simulated explosion field

**Figure 2:**
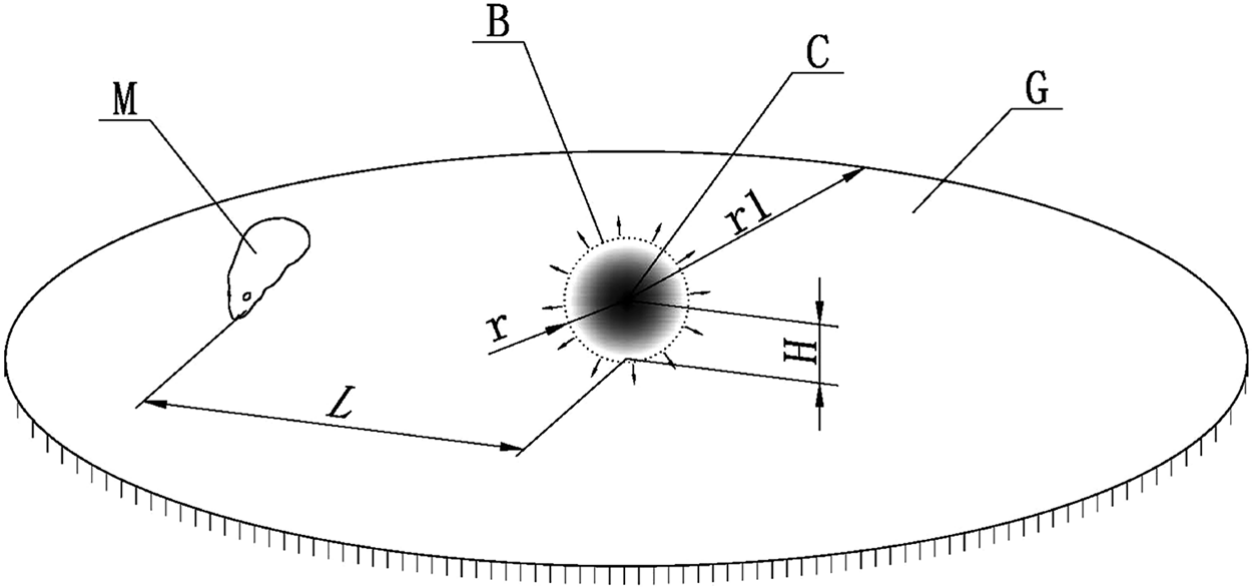
The Explosion Field (the spherical radius r = 10 cm of the initial wave, M refers to the rat, H = 10 cm, r1 = 2 m, L refers to the distance between the explosion center and the rat).The detonation force was an explosive force selected equivalent to a 10 g TNT charge with a spherical radius r = 10 cm of the initial wave of the shock wave.

We defined the explosion field as in Fig. 2. For the establishment of the explosion field, a 2 m by 2 m by 2 m cuboid air space was set as boundary areas where the blast wave propagated. The 3D rat was separately set at 40 cm, 50 cm 60 cm, 70 cm and 80 cm from the denotation center. The explosion wave could only proceed from one point to a spherical shape. The simulated blast field contained an initial wave of 10 cm in a spherical shape with an explosion center C at the center of the sphere. The simulated explosion field also included a simulated ground of 200 cm at the bottom of the boundary areas. The distance H of the explosion center from the simulated ground and the spherical radius r of the initial wave of the shock wave were also included.

In this simulation, the detonation force was selected equivalent to a 10 g TNT charge. with a spherical radius r = 10 cm of the initial wave of the shock wave. We calculated the initial pressure force in the initial wave of 10 cm according to the calculation formula of the explosive initial pressure conversion: P = 0.084/R + 0.27/R^2^ + 0.7/R^3^ (R = r/W^1/3^), where R is the proportional distance, which is the ratio of the distance r (m) of the explosion center to one-third square of the explosive dose W (kg). W was calculated by the TNT equivalent. P was the shock wave pressure in kPa.

In this experiment, after the calculation of the formula, the pressure value of the explosive pressure at the initial wave was set to be 8442 kPa as 10 g TNT equivalent. The calculation process started from the initial shock wave.

### Calculation process

The calculation process consisted of two parts. The first part involved computational air fluid dynamics, which was used to mimic the process of shock wave moving from the start of the shock wave to the surface of the animal. The wave started from the explosion center, went through the air and had an impact on the surface of the animal.

After establishing the simulated explosion field and selecting the turbulence model, we defined the calculation of the domain fluid as the ideal gas, which accorded with PV = nRT (P is the air pressure, V is the volume, n is the amount of substance of the gas, R is the gas constant 8.314 J·K^−1^·mol^−1^, T is the absolute temperature). The surfaces were defined as solid wall without slipping effects. Before the explosion, the space air pressure of the calculated area was set to 101.325kPa and the initial ambient temperature condition was set to 25 degrees Celsius. The calculation process was set as the process of calculating transient air fluid. The convergence criterion was set as 10^−4^. The established explosion field and the surface of the 3D rat model were all imported into the CFD software FLUENT (ANSYS, Canonsburg, PA) to complete the simulation process. Every step of air fluid dynamics calculation and analysis made by the FLUENT software was based on the law of the conservation of mass, momentum and energy, which was applied to the solution of the velocity components, pressure and temperature.

The whole simulation process involved a pressure distribution of 1.5 s. The initial pressure was set to be increased linearly as the shock wave started. At the time of t = 0 s, the initial pressure at the explosion was set as zero. Gradually, the explosive center added its pressure to a peak of 8442kPa at the time point of 750 ms. It declined to 0kPa as the simulation ended at the time point of 1.5 s. This process contained the process of overpressure, which is the main cause of lung injury. The pressure of the rat surface was measured when the pressure came to its peak. The surface pressure could then be validated by real experiments.

The second part involved a series of structural analysis of the mechanics. When the shock wave arrived at the surface of the animal, it penetrated the skin, soft tissue, muscles and the rib cage and caused injuries to body structures. This simulation contained this process. In this simulation, a linear static structure pressure analysis of the lung was performed.

All the components of the structures were imported into ANSYS Workbench (ANSYS, Canonsburg, PA) for further analysis. After receiving the structures and the initial conditions before calculation, the data was calculated in the ANSYS workbench software. Then, the data from the ANSYS software was transferred to the workbench software in order to illustrate the pressure distribution on the surface of the rat and lung pressure. In the simulation, we put the initial conditions of the mechanics the same as the surface pressure distribution of the previous simulation. We assumed that K (the stiffness coefficient matrix) was continuous and that the corresponding materials satisfy the linear elasticity. In the software setting, we conducted the calculation by the software according to the general equation for the mechanical dynamics of an object^23^ as: $M\ddot{x}+C\dot{x}+{Kx}=F(t)$. In the formula, $M$ is the mass matrix, *C* is the damping matrix, $K$ is the stiffness coefficient matrix, $x$ is the displacement vector, $\dot{x}$ is the velocity vector, $\ddot{x}$ is the acceleration vector and $F(t)$ is the force vector.

**Figure 3:**
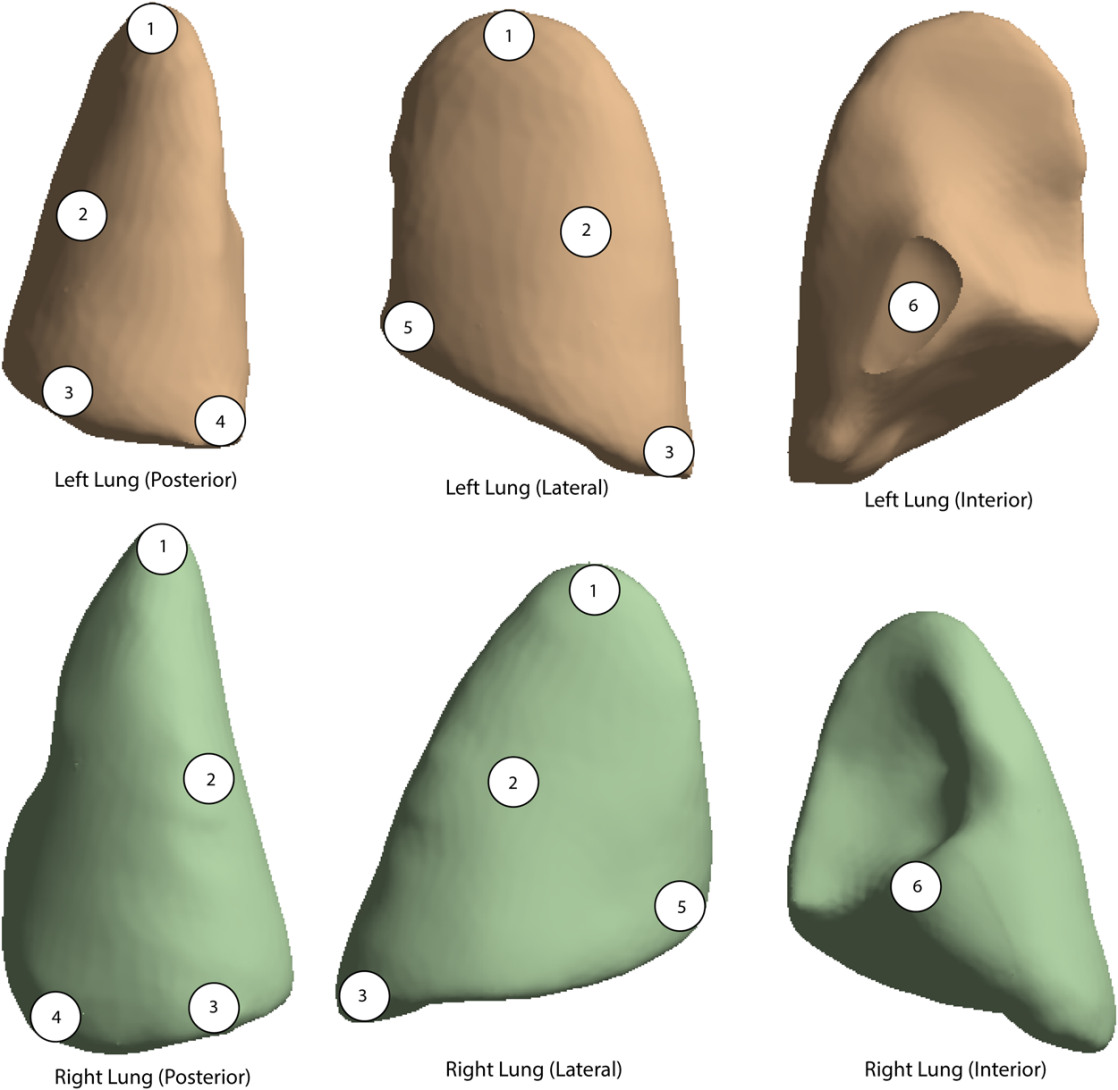
6 points from both sides of the lung tissue are selected for pathological biopsy. Point 1–6 represented apex pulmonis, middle lateral lung, lower lateral lung, lower interior lung, lower anterior lung and interior lung, respectively.

In order to monitor the mechanical responses of the lungs to blast waves, 6 virtual points are placed on the surface of each lung lobe for statistics (Fig. 3). Pathology experiments were then conducted to validate the pressure distribution of the lung.

### Validation

36 Male Sprague-Dawley rats weighing between 220 g and 290 g from the Academy of Military Medical Sciences (Beijing, China) were chosen. The animals were kept in animal containers at environmental conditions including a temperature of 25 °C and a relative humidity of 60% for 7 days. All the rats were anaesthetized before the injuries. The rats were then autopsied for pathological observation. Blast injuries were inflicted on the animals by the source of the explosion, which was a compressed hexogen stick equivalent to 10 g TNT. In the experiments, the left chest was set to face the source of explosion. This experiment was authorized by the Animal Ethics Committee of the First Affiliated Hospital of PLA General Hospital. All methods were performed in accordance with the relevant guidelines and regulations.

In the experiment, the rat model is located on the ground, set at a distance from the explosion center L = 40 cm, 50 cm, 60 cm, 70 cm, 80 cm. The data of the surface pressure was recorded at the 5 distances. 5 injured groups contained 30 rats, 6 rats for each group respectively. The remaining 6 rats with no blast injury were used as an untreated group for pathological scoring. The surface pressures of animals were recorded by sensors (Pressure transducer model 113A21, PCB company).

The simulated pressure values obtained using the finite element analysis method were also recorded. In order to verify the accuracy of the simulated data, corresponding animal experiments were carried out to verify that each explosion experiment. After the explosion, the pathological results were performed to validate the pressure distribution of the lung injury.

In each lobe, 6 points from the lung tissue were selected for pathological biopsy in order to validate the injuries caused by pressure. From pathological observations, an expansion and movement of the alveoli and capillaries could be observed fully. A rupture of the alveolar walls and capillaries, blood leakage, pulmonary microvascular extensive gas and a fat embolism were also discovered.

Smith scoring was performed according to the pathological findings. The scoring was conducted after HE staining by 3 experienced pathologists at the same time to get an average recorded score. The lung pathology was assessed by being scored on a 0–4-point scale (0 points represents no injury; 1 point reveals 25% of the scope field; 2 points reveals 50% of the scope field; 3 points reveals 75% of the scope field; 4 points reveals over 75% of the field). The conditions of the lung edema, hemorrhage and inflammation were each scored to stack up to a total score.

By using SPSS 17.0 (SPSS Inc., Chicago, USA), Pearson’s test was performed for a comparison between the pressure values and average pathological Smith scores of every point at the 5 distances. 1 represented a total positive linear correlation, 0 represented no linear correlation, and −1 represented a total negative linear correlation. Paired T test was conducted between the Smith scorings of the injured group at the 5 distances and the untreated groups.

## Results

The predicted surface pressure and lung von Mises stress were simulated for the prediction of the lung injury for a given blast wave loading. The blast overpressure had an evident impact on the lung surface. The blast wave caused a significant pressure peak when it arrived at the thorax and abdomen areas of the rats. The wave penetrated through peripheral structures of the lung tissue and caused pressure peaks on the lung surface. We compared the measured pressure peaks of the five distances to the simulation pressure peak. The distribution patterns of the body surface and lung surface pressure were simulated to estimate the severity of injury in different lung lobes. The pathological results validated the severity distribution.

### Simulation Results of the Body Surface Pressure

The three-dimensional model of the rat was set at distances of 40 cm, 50 cm, 60 cm, 70 cm and 80 cm, respectively. The corresponding maximum pressure of the surface was 634.77kPa, 362.46kPa, 248.11kPa, 182.13kPa and 109.29kPa. The further away the rat chest was from the site of explosion, the maximum pressure exerted on the chest wall was reduced.

**Figure 4:**
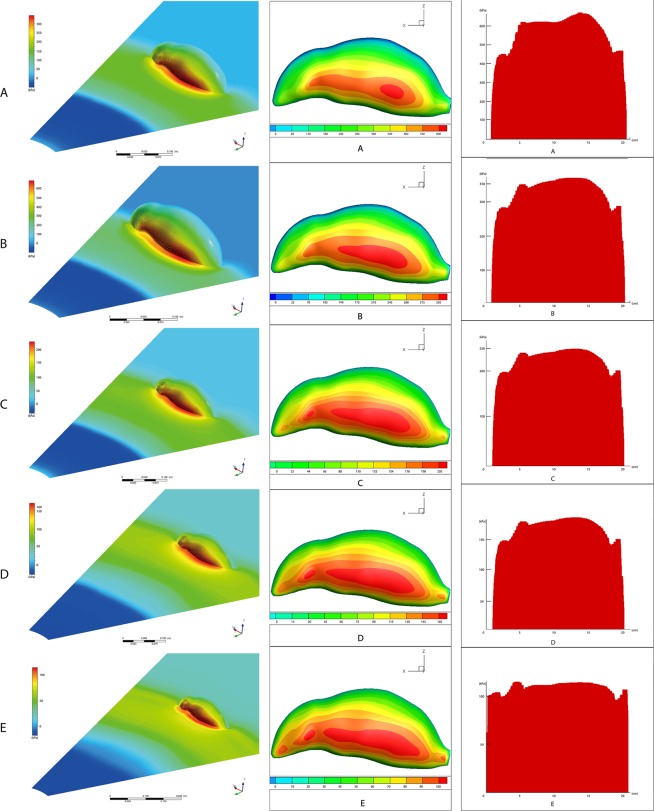
Simulation Results of the Body Surface Pressure (**A**): 40 cm; (**B**) 50 cm; (**C**) 60 cm; (**D**) 70 cm; (**E**) 80 cm; LEFT: Explosive view; MIDDLE: rat surface view; RIGHT: pressure distribution from the HEAD to the AFTERBODY).

The simulation results suggest that the maximum pressure rises to 595kPa from the head to the neck when the blast center is 40 cm away from the rat. In the chest area, the pressure reached a maximum of 586kPa at a distance of 70 cm from the top of the head and then decreased. In the abdomen area, the pressure reached 631kPa, followed by a continued decrease in pressure to the tail part (Fig. 4A).

When the blast center was 50 cm, 60 cm and 70 cm away, the pressure distribution showed the same trend. From head to neck, the maximum pressure increased to 344kPa, 239kPa and 176kPa, respectively. In the chest area, the pressure reached the maximum at 7.5 cm, 8.0 cm and 8.5 cm from the head top, respectively, which was 273kPa, 191kPa, 149kPa, and then decreased. In the abdomen area, the pressure increased to 359kPa, 248kPa and 182kPa, and the pressure in the caudal area continued to decrease (Fig. 4B–D).

When the center of the explosion was 80 cm from the head to the neck area, the maximum pressure value increased from 0kPa to 108kPa, the maximal pressure change in the chest area increased slightly to 109kPa and then remained stable to the abdomen area before reaching a maximum in the abdomen area 110kPa. The farther away from the head, the lower the pressure value (Fig. 4E).

### Simulation Results of the Lung Surface Pressure

**Figure 5:**
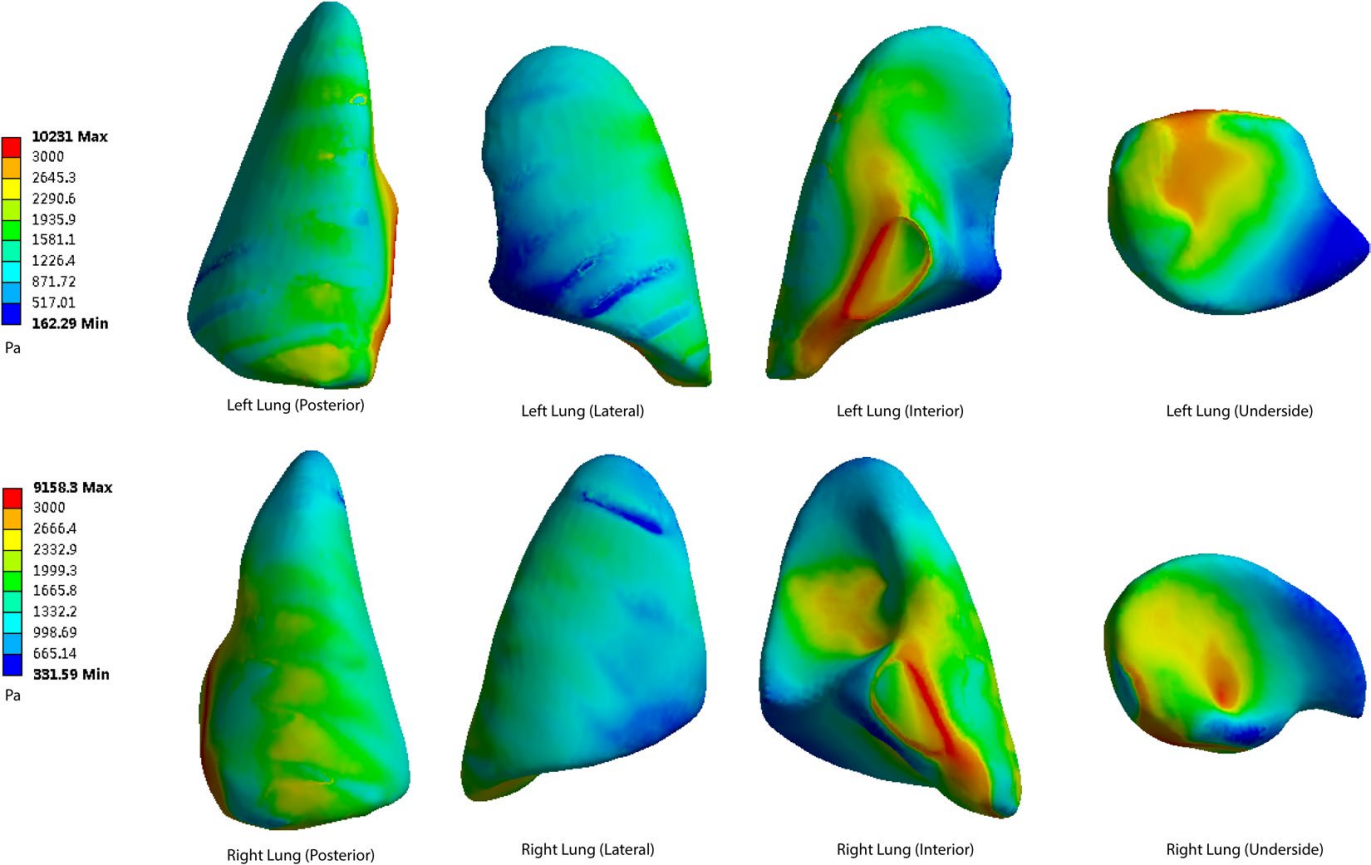
The pressure peaks at the posterior, lateral, interior and underside of the lung surface (40 cm). The interior and underside of the lung surface have higher levels of von Mises pressures. The apex pulmonis has the lowest shear stress. Blocking effect can be observed. Warm color represents high pressure area, cool color represents low pressure area(40–80 cm). Maximum pressure was 10231 Pa.

**Figure 6:**
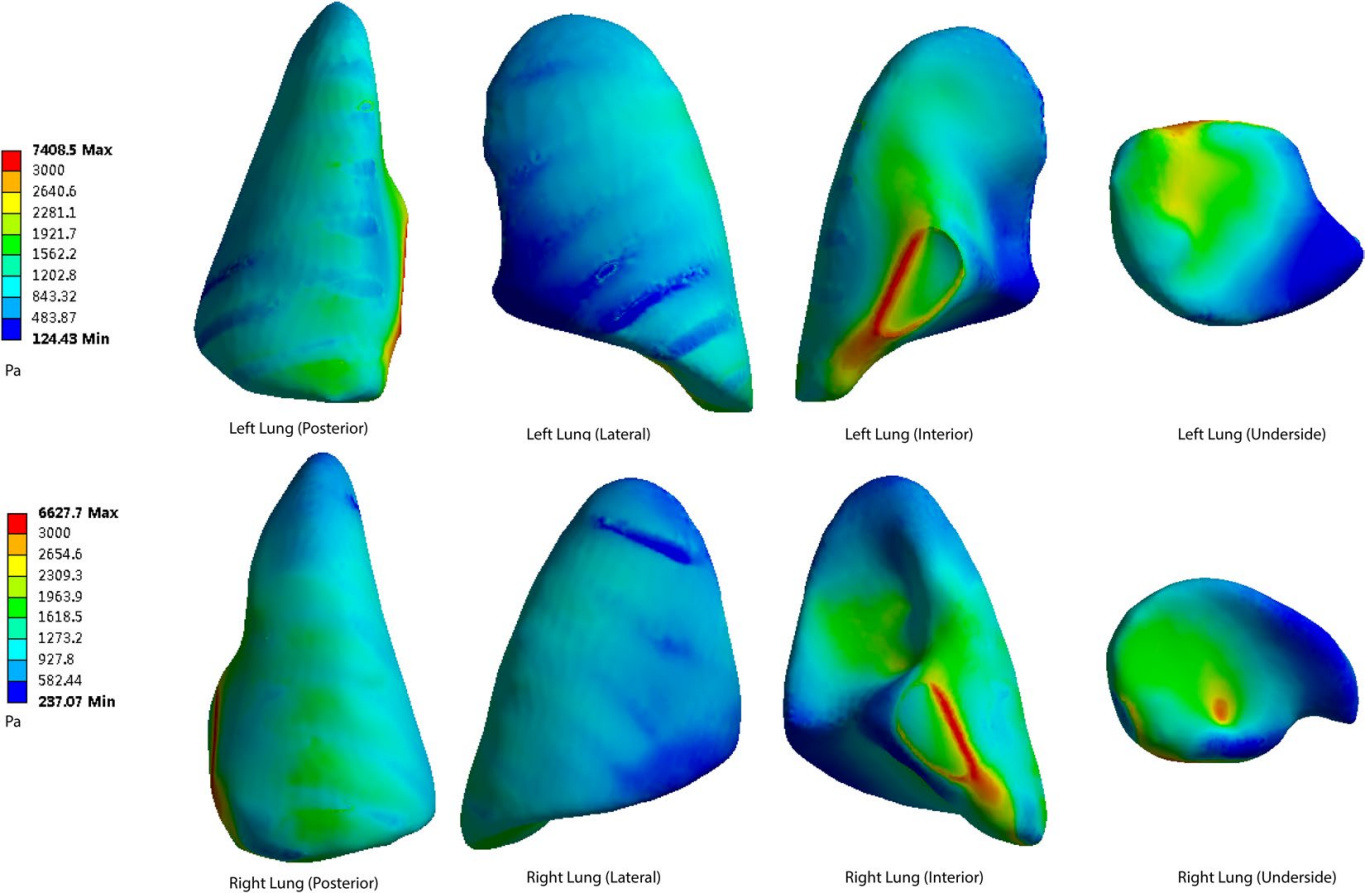
The pressure peaks at the posterior, lateral, interior and underside of the lung surface (50 cm). Maximum pressure was 7408 Pa.

**Figure 7:**
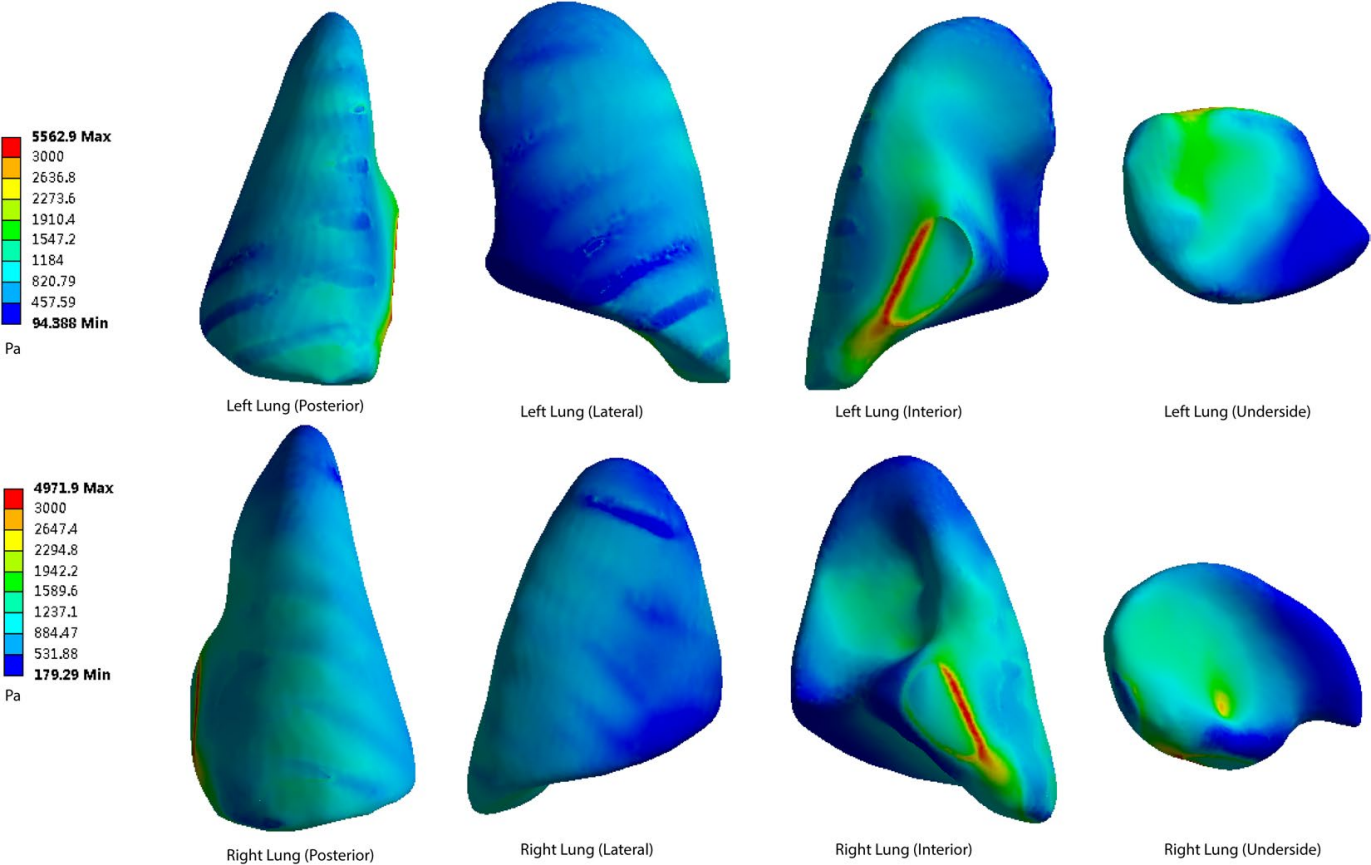
The pressure peaks at the posterior, lateral, interior and underside of the lung surface (60 cm). Maximum pressure was 5562 Pa.

**Figure 8:**
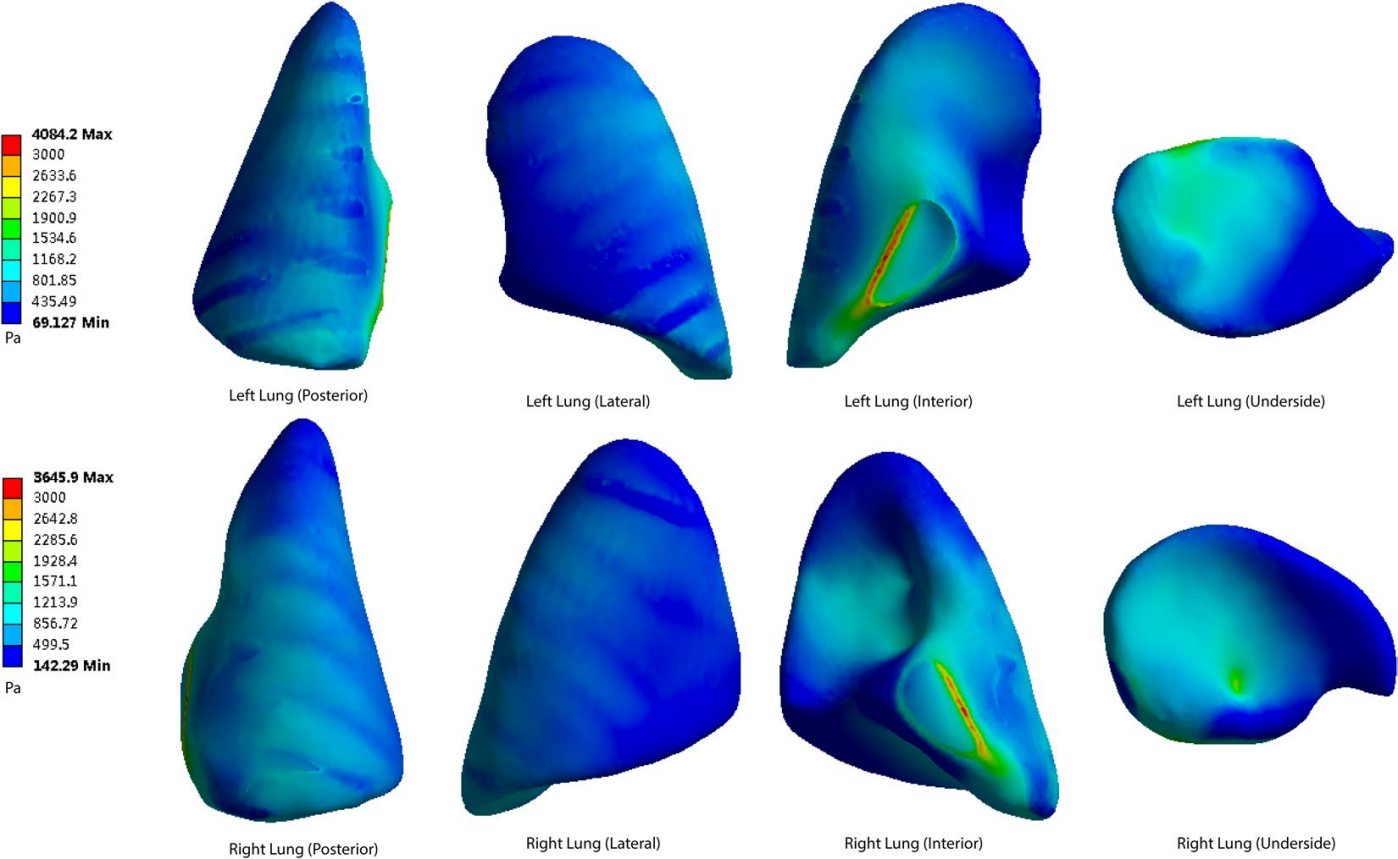
The pressure peaks at the posterior, lateral, interior and underside of the lung surface (70 cm). Maximum pressure was 4084 Pa.

**Figure 9:**
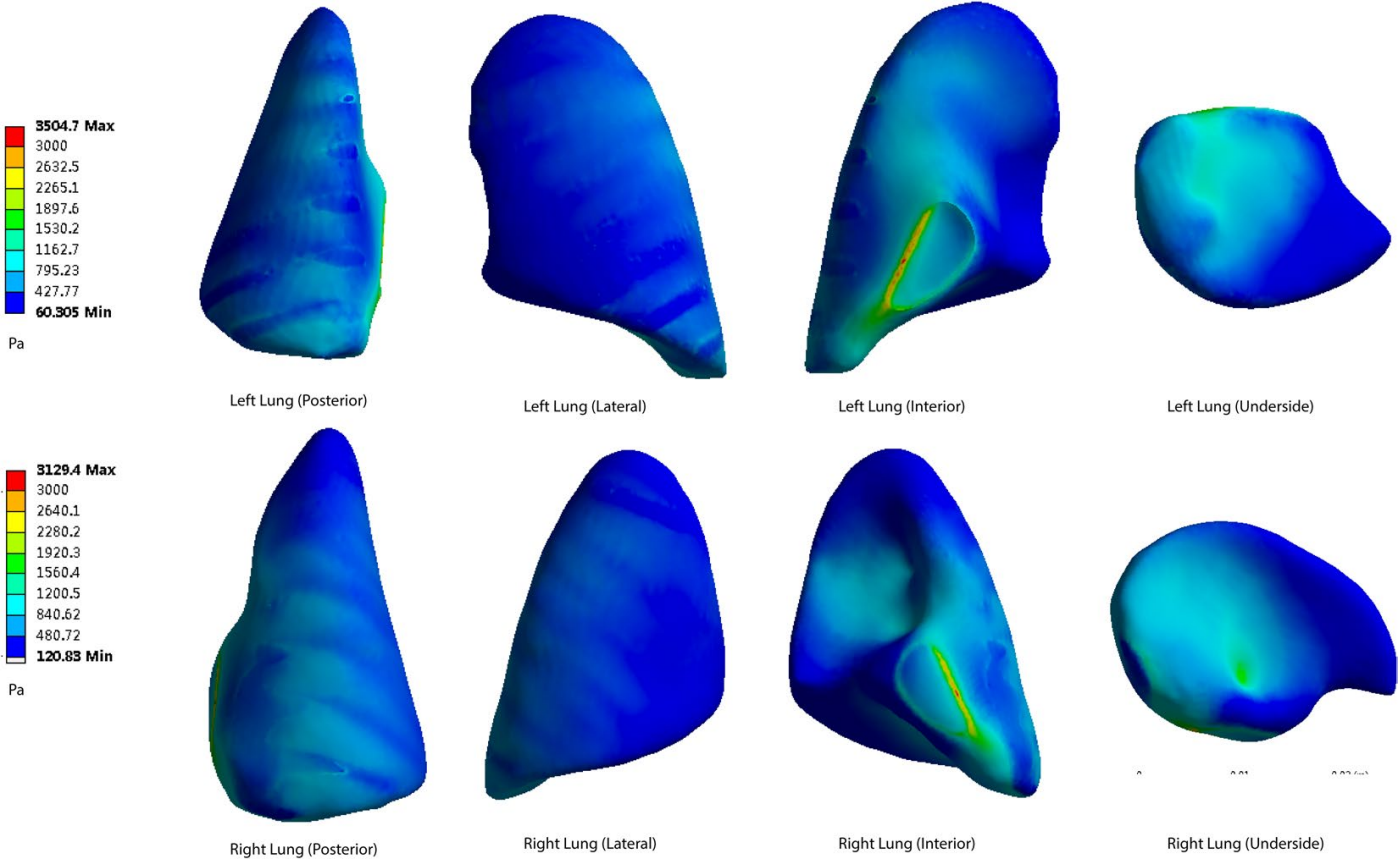
The pressure peaks at the posterior, lateral, interior and underside of the lung surface (80 cm). Maximum pressure was 3504 Pa.

The shock wave penetrated the rat’s body, inducing pressure on the surface of the lung. The pressure peak was simulated in the figures. In this part of the simulation, the rats were set separately at 40 cm, 50 cm, 60 cm, 70 cm and 80 cm from the center of the explosion. The von Mises pressure peaks at the posterior, lateral, interior and underside of the lung surface were measured and recorded as Figs 5–9.

When the distance from the explosion increases, the pressure levels become lower at every part of the lung surface. At the posterior side of the lungs, the lowest pressure occurs at the apex of the lung. The underside of the lung has a higher pressure. On the lateral side, the top area shows a lower pressure than the bottom area. The interior and underside of the lung surface are found to have higher levels of shear pressures than other areas, and the apex pulmonis has the lowest shear stress. On the posterior and lateral sides of the lung surface, the ribs have a blocking effect, meaning that pressure distribution level fluctuates when the stress penetrates the ribs.

The highest pressure monitored on both sides of the lungs is on the medial side of the lung surface. The extreme pressure values on the left lungs at the five distances are 10231 Pa, 7408 Pa, 5562 Pa, 4084 Pa and 3504 Pa, respectively. The extreme pressures on the right lungs are 9158 Pa, 6627 Pa, 4971 Pa, 3645 Pa and 3129 Pa. In the posterior scenario, the pressures of both sides of the lungs range from 928 Pa to 2916 Pa when the distance is 40 cm (Fig. 5). When the distances are 50 cm, 60 cm, 70 cm and 80 cm, the pressure ranges are 733–2254 Pa, 488–1236 Pa, 357–1189 Pa and 314–992 Pa (Figs 6–9).

6 points of the simulated surface pressure were monitored at the 5 distances. The points represent the edges of the lungs. Point 1–6 represented apex pulmonis, middle lateral lung, lower lateral lung, lower interior lung, lower anterior lung and interior lung, respectively. The surface pressures of the 6 points are shown in Table 1.

**Table 1:** 6 points of the surface pressure were monitored at the 5 distances(40–80cm).

The Surface Pressure of Lung Injury (Points 1–6, Left and Right)							
		1	2	3	4	5	6
40 cm	L	1110	1297	1350	2322	438	2751
	R	928	1374	1365	2916	736	2240
50 cm	L	795	949	1049	1924	289	2009
	R	733	1023	1056	2254	512	1565
60 cm	L	598	673	715	950	204	1466
	R	488	765	725	1236	355	1177
70 cm	L	437	511	529	933	168	1026
	R	357	566	568	1189	305	922
80 cm	L	379	409	309	668	141	982
	R	314	480	478	992	244	756

### Validation

**Figure 10:**
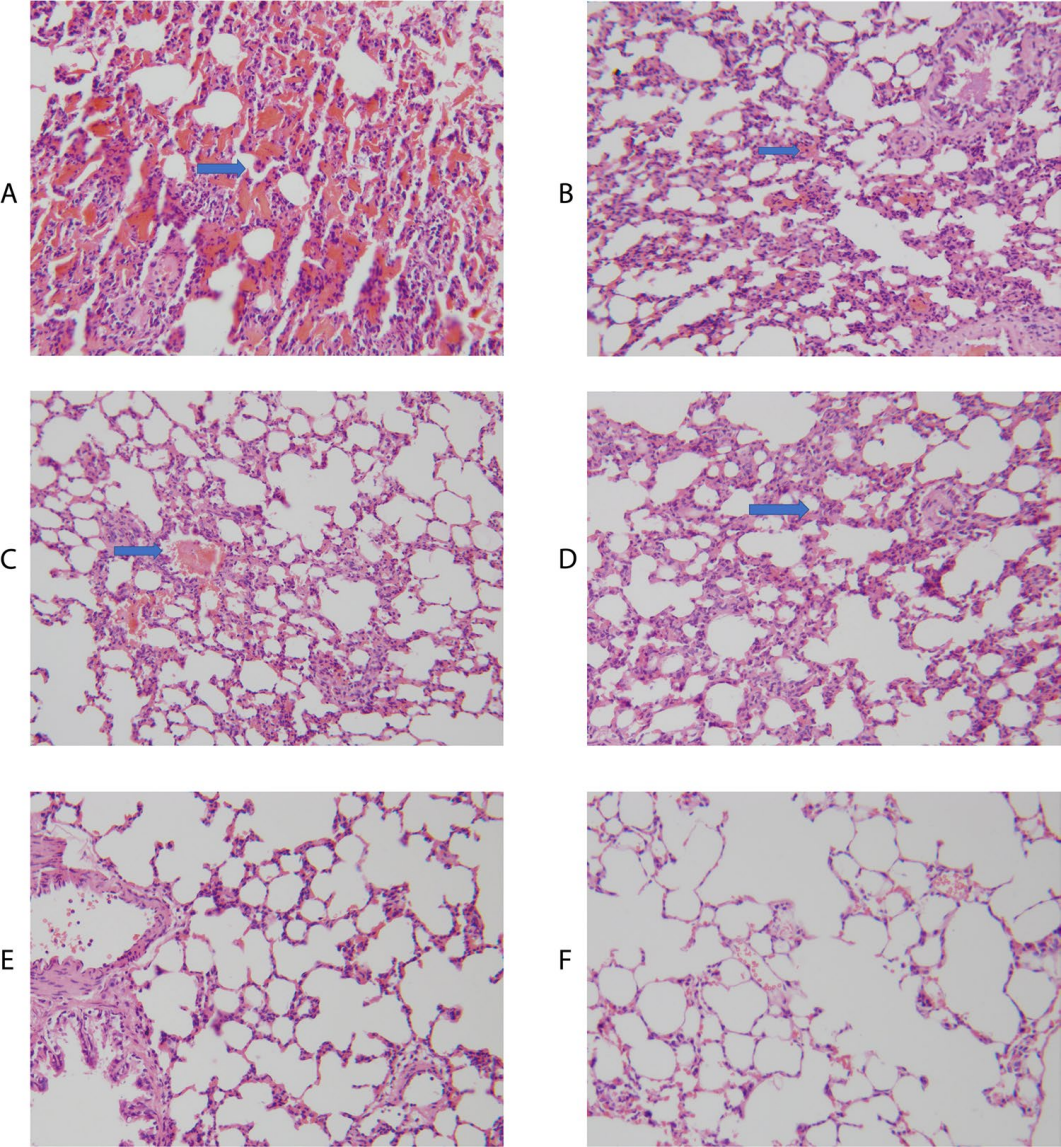
The pathology observations of the 5 distances at Point 6 (**A**) 40 cm, an accumulation of alveolar interstitial fluid and blood and necrosis of the alveolar; (**B**) 50 cm, the edema and alveolar bleeding; (**C**) 60 cm, mild edema and bleeding; (**D**) 70 cm, mild edema and bleeding; (**E**) 80 cm, almost normal alveoli; F: Untreated Group, no injury found).

The tracheas were clamped immediately when the lung tissue was aerated. A midline sternotomy was performed on each rat and both sides of the lung tissues were then harvested with the tracheas closed. The 6 points on the lung tissues were embedded precisely and separately in paraffin and HE stained obtained after the injury. When the rat was 40 cm from the explosion center, large areas of edema, an accumulation of alveolar interstitial fluid and blood and necrosis of the alveolar were discovered (Fig. 10A). When the distance was moved to 50 cm, the edema and alveolar bleeding were still discovered (Fig. 10B).

On the surface of the lungs, hemorrhagic spots could be discovered. Most of them were found at the bottom of both sides of lung tissue. When the distance was 60 cm, few hemorrhagic spots could be found and there were still mild lung edemas and an accumulation of interstitial fluid (Fig. 10C). At distances of 70 cm and 80 cm, few accumulations of fluid were discovered and most of the lung surface was normal (Fig. 10D,E).

Pearson correlation coefficient between the surface pressure of lung injury and the pathological scores at the 5 distances were measured. For the left side of the lung, when the distance was 40–60cm, the Pearson’s values were 0.899, 0.949 and 0.939, which reflected the correlations between the simulation pressure and the injury. For the right side of the lung, the Pearson’s values were 0.891, 0.908 and 0.941, which also showed strong relations. However, when the distances were 70 cm and 80 cm, the Pearson’s values were less than 0.80 except for the right lung at 80 cm, which showed weak linear correlations. For both sides of the lung, when the distance was 40cm-80cm, the correlations between injured groups and untreated group were significant. When the distance was 80 cm, the correlation was not significant (Table 2).

**Table 2:** Pearson correlation coefficient between the surface pressure of lung injury and the pathological scores (SPSS Inc., Chicago, USA, 17.0).

		1	2	3	4	5	6
40 cm	L	5	6.8	10	12	4.6	11.8
	R	4.4	6.2	9	11.6	6	9.2
50 cm	L	3.4	4.6	6.6	8.4	3.2	9
	R	2	3.4	5.6	8.2	2.2	8
60 cm	L	2	2.2	3.8	5.4	1.6	7.4
	R	1.4	3.4	3.6	4.8	2	5
70 cm	L	1.2	1.8	2.4	3.8	2.6	3
	R	0.8	2	1.4	2.6	1.8	3
80 cm	L	0	0.2	0.2	0.6	0	0.2
	R	0	0	0	0.6	0	0.2
Untreated Group	L	0	0	0.1	0.3	0	0.2
	R	0	0.2	0	0.3	0	0.2
		Pearson Correlation Coefficient		Pearson Correlation Significance		T-test Significance with the Untreated Group	
40 cm	L	**0.899**		**0.015**		**0.001**	
	R	**0.891**		**0.017**		**0.001**	
50 cm	L	**0.949**		**0.004**		**0.002**	
	R	**0.908**		**0.012**		**0.007**	
60 cm	L	**0.939**		**0.005**		**0.009**	
	R	**0.947**		**0.004**		**0.002**	
70 cm	L	0.604		0.204		**0.001**	
	R	0.783		0.605		**0.001**	
80 cm	L	0.498		0.314		0.111	
	R	**0.912**		**0.011**		0.809	

The lung pathology was assessed by being scored on a 0–4-point scale (0 points represents no injury; 1 point reveals injury in 25% of the scope field; 2 points reveals injury in 50% of the scope field; 3 points reveals injury in 75% of the scope field; 4 points reveals injury in over 75% of the field). The conditions of the lung edema, hemorrhage and inflammation were each scored to stack up to a total score. Pearson’s test was performed for a comparison between the pressure values and average pathological Smith scores scores of every. Point at the 5 distances. 1 represented a total positive linear correlation, 0 represented no linear correlation, and −1 represented a total negative linear correlation. Paired T test was conducted between the Smith scorings of the injured group at the 5 distances and the untreated groups.

## Discussions

In this study, a three-dimensional structure of rat tissue was established through finite element modeling. We used this structure to simulate the conditions of an impact force. The lung tissue was surrounded by rib bones, muscles and skin. The surface pressure distributions of skin and lung tissue were simulated based on the 3D structure.

The explosion center is surrounded by air. The atoms that fly at a high speed of act on the air closest to the center of the explosion. This impact not only pushes this layer of air away from the center of the nuclear explosion but also transfers the explosive energy to it. This impact transmission process produces a movement of air propagating to the outside, and the impact pressure is enough to cause the air to move faster than the speed of sound. The spherical shock wave front and the impact wind with a specific pressure are emitted from the explosion center, hit the ground and the ground blocks the shock wave. As a result, a portion of the spherical shock wave reflects back from the ground and enhances the enormous overpressure that is formed. The reflection process of shock wave reflection was observed and simulated in the calculation process.

The shock wave front approaches and impacts the target surface, exerting a dynamic pressure, and the target receives an impact. When a shock wave reaches an object, it produces two effects. The first is the squeeze effect of static overpressure. If the target is a closed object, it will suffer crush damage^24^. The second damage effect is called the drag load. It is a force generated when the impact wind is blown to the opposite side of the explosion center, which drags the target toward the opposite direction as a whole^25^.

The peak static overpressure of the shock wave decays with time and a certain degree of negative pressure can be formed in the later stage. The negative pressure phase lasts longer than the positive pressure phase, but its static pressure and dynamic pressure are much lower than the positive pressure phase^1^. In the negative pressure phase, the object obtains a lower pressure, resulting a lower possibility of receiving damage than positive pressure phase. Therefore, we chose to observe the peak value of the shock wave as the main observation to judge the pressure distribution.

When explosives are detonated, the object is near the explosion point, causing tears in the skin and internal organs, then the parts of the body are seriously displaced. The shock wave has an influence on the activity of gas during the traveling process.Gas-containing organs such as the lungs, the middle ear and the gastrointestinal tract are most likely to receive the shock wave, resulting in overpressure damage. Pathological manifestations include direct lung injury, tympanic membrane rupture, intestinal contusion and intestinal perforation. Blood leakage and an expansion of the alveoli and capillaries occur after lung injury. Oxidation cascade amplification also played a role in respiratory epithelial cell apoptosis^10^. According to the simulation results, at the surface of the skin, a pressure peak occurred at the chest area or abdomen area. The simulation results showed that pressure distribution could also play a role in lung and abdominal organ damage.

When the pressure penetrated the surface of the lung, the interior and underside of the lung surface showed higher shear pressures while the apex pulmonis showed a lower shear stress. The validation results reflected that at both sides of the lung at distances of 40–60cm, the pathological scores and the pressure levels showed strong correlations. The distances of 70 cm and 80 cm showed weak correlations. The pathological scores were collected 6 hours after injury. Both the pathological results and simulation results showed that the interior and underside of the lung are more susceptible to blast injury. Since a higher peak pressure can squeeze and drag organs and cause damage, the interior and underside high pressure might induce high pathological scores. Gravity can also cause the exuded fluid to move in the lungs, resulting in the accumulation of lower lung fluid, increased density and an increased pathological score. When the distance was 70 cm, peak pressure declined sharply and the damage was mild. The pathological results also showed little exuded fluid accumulation. The correlation between the scores and simulation pressures was not so evident at 70–80 cm.

The mathematical model of human trauma began in the mid-1970s^26^. In 1988, Stuhmiller *et al*. proposed a damage biomechanical model related to spring damping systems. Various chest injury models have various impact injuries, non-punch-through impacts and blunt injuries^27^. This model greatly simplified the geometry of the lungs, ignoring the details of internal wave propagation, internal organ interactions and the viscoelasticity of the thoracic itself, calculating the average energy density of the whole lung and providing a three-dimensional distribution of high energy in the lungs. The analysis of the motor car collision is presented In 2002, Iwamoto *et al*. demonstrated a complete human model of collision safety^28^. More relevant computer models are now used in related research by estimating the energy distribition^27,29,30^. Compared to previous studies, this simulation provided a more specific view of pressure distribution, validated a relationship between injuries and pressure distribution by rat explosion experiments. The actual explosion and validation process are also added in order to investigate the effectiveness of this method. This computational simulation provided dynamic anatomy as well as functional and biomechanical information. However, the constants of every step of simulation were fixed and the animal sizes could be slightly different. There might be some bias between the actual explosion and the simulation explosion if there were minor environmental state changes. Due to the complex structure of the alveoli and the difficulty of standardizing the boundary conditions, the alveolar level of simulation is still difficult. The establishment of the models help to enhance the understanding of biomechanical pressure wave propagation and help determine the extent of primary lung injury. According to previous studies, this modelling could be applied to evaluate the density levels of the lung tissue after blast injury at different timepoints. Other factors that may cause fluid in the lungs might also be investigated by testing lung density. In this simulation, higher levels of pressure showed more severe injury. From the validation of pressure levels and actual pathological results at certain degrees of pressure, based on estimated pressure levels or distribution, we are able to evaluate the severity of injuries. Subsequent research has expanded on the use of three-dimensional reconstruction technology to simulate a variety of explosion scenarios such as mines, mortars, grenades and a variety of modified explosives. Certain protective methods could also be added to future simulation to evaluate the blocking effects. Human body simulations with and without protective armor are also a future intention for investigation in future experiments.

## Supplementary information

Table 1 and Table 2
