# Author Correction: Simulation of blast lung injury induced by shock waves of five distances based on finite element modeling of a three-dimensional rat

**DOI:** 10.1038/s41598-020-76580-7

**Published:** 2020-11-09

**Authors:** Chang Yang, Zhang Dong-hai, Liu Ling-ying, Yu Yong-hui, Wu Yang, Zang Li-wei, Han Rui-guo, Chai Jia-ke

**Affiliations:** 1 Department of Burn and Plastic Surgery, Burns Institute, Burn & Plastic Hospital of PLA General Hospital, Fourth Medical Center of PLA General Hospital, Beijing, 100048 PR China; 2 Science and Technology on Transient Impact Laboratory, Beijing, 102202 PR China

Correction to: *Scientific Reports* 10.1038/s41598-019-40176-7, published online 05 March 2019

This Article contains an error in the Methods section, under the subheading ‘Establishing the simulated explosion field’.

“P was the shock wave pressure in kPa.”

should read:

“P was the shock wave pressure in MPa.”

